# KReach: A Tool for Reachability in Petri Nets

**DOI:** 10.1007/978-3-030-45190-5_22

**Published:** 2020-03-13

**Authors:** Alex Dixon, Ranko Lazić

**Affiliations:** 8 Johannes Kepler University, Linz, Austria; 9 University of Birmingham, Birmingham, UK; Department of Computer Science, University of Warwick, Coventry, United Kingdom

**Keywords:** Petri Nets, Kosaraju’s Algorithm, Reachability, Vector Addition Systems, Coverability

## Abstract

We present KReach, a tool for deciding reachability in general Petri nets. The tool is a full implementation of Kosaraju’s original 1982 decision procedure for reachability in VASS. We believe this to be the first implementation of its kind. We include a comprehensive suite of libraries for development with Vector Addition Systems (with States) in the Haskell programming language. KReach serves as a practical tool, and acts as an effective teaching aid for the theory behind the algorithm. Preliminary tests suggest that there are some classes of Petri nets for which we can quickly show unreachability. In particular, using KReach for coverability problems, by reduction to reachability, is competitive even against state-of-the-art coverability checkers.

## 1 Introduction

Petri nets [26] (equivalently, Vector Addition Systems with States [12,14]) are one of the best-known formalisms in concurrency theory. They form a highly expressive model which is applicable in a broad range of domains including software and hardware verification [5,6], chemical modelling [3], and business processes [22]. Two of the most studied decision problems on Petri nets are those of *coverability* and *reachability*.

Coverability is the central decision problem for verifying safety properties on Petri nets. The coverability problem asks, given a starting configuration $\mathbf{m_0}$ and a target $\mathbf{m}$, whether we can reach, by some sequence of valid transitions (i.e. by a *run*), any configuration $\mathbf{m}' \geq \mathbf{m}$. The problem is known to be EXPSPACE-complete [23,27]. Coverability has seen considerable study in recent years, in particular with a view towards minimising the running time of coverability decision procedures [2,11].

The reachability problem, to which coverability is easily reducible, can capture both safety and liveness properties of systems [13]. Formally, the reachability problem asks if we can, by some run, get from a starting configuration $\mathbf{m_0}$ to the target configuration $\mathbf{m}$ exactly. Historically, there has been a wide gap

* Supported by the Centre for Doctoral Training in Urban Science and Progress (EPSRC Grant EP/L016400/1).

between the upper and lower bounds, but recently these have been improved to a non-elementary lower bound [7] and an Ackermannian upper bound [21].

The first complete algorithm for reachability is due to Mayr [24], since further developed and simplified by Kosaraju [17] and Lambert [18]. More recently, a strikingly simple but not yet practical algorithm was obtained by Leroux [20], based on enumerating Presburger-definable invariants. In this paper we will focus on Kosaraju's algorithm. The latter is the subject of an entire book by Reutenauer [29], since translated into English [28], and more recently presented in a novel, readable format with contemporary notation by Lasota [19].

In spite of these substantial and sustained theoretical developments, the area has seen little in the way of practical implementation. We seek to address this gap by making the following contributions:

- We present a tool, KReach, which we believe to be the first complete implementation of a decision procedure for the general Petri net reachability problem.
- Noting that the reachability problem is infamous for its complexity—both in terms of its worst-case runtime, and the impenetrability of its decision procedures for newcomers—we offer an accessible implementation of Kosaraju's algorithm, which can be used as a detailed learning aid.
- We have designed the implementation in a modular and extensible way which is conducive to development of future improvements to the algorithm.
- We include a number of parameterised example nets which demonstrate correctness and performance, and can be used to assess further work.
- We provide a full suite of libraries which aid programming with Vector Addition Systems (with States) in the Haskell programming language.

## 2 Design and Implementation

**Algorithm** We will now give a brief overview of Kosaraju's classic reachability algorithm for Petri nets, and explain the translation into code. Note that Kosaraju's algorithm operates over VASS—the translation between the two is immediate, and can be performed while parsing the problem instance.

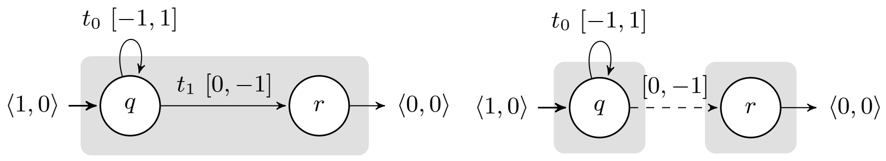

(a) We denote ⟨constraints⟩ and [transitions]. The states of our original VASS are $q$ and $r$; the transitions are $t_0$ and $t_1$. Here we are testing reachability from $q(1, 0)$ to $r(0, 0)$.

(b) $\mathcal{G}_{\text{ex}}$ after SCC decomposition. We now have two components, the second of which is trivial. The adjoinment is marked by a dashed arrow. The shaded rectangles are separate components.

Fig. 1: A simple GVASS, $\mathcal{G}_{\text{ex}}$.

The procedure revolves around *Generalized VASS (GVASS)*, an extension of VASS. A GVASS $\mathcal{G}$ is a sequence $(C_1, .., C_n)$, of VASSs annotated with metadata, most notably constraints on their entry and exit configurations. The exit state of $C_i$ is adjoined to the entry state of $C_{i+1}$ by a transition. Reachability in our original VASS $V$ is implied by reachability in an induced GVASS $\mathcal{G}(V)$, a singleton sequence where we constrain the entry and exit configurations as being equal to our initial $\mathbf{m_0}$ and target $\mathbf{m}$. Figure 1a gives an example.

In outline, Kosaraju's algorithm operates as follows. At each step, given a strongly-connected GVASS $\mathcal{G}$:

- either $\mathcal{G}$ fulfils $\theta$;
- or $\mathcal{G}$ violates $\theta$, in which case we can *refine* $\mathcal{G}$ into a finite (possibly empty) set of modified GVASSs.

This produces a finitely-branching tree of GVASSs in which every branch forms a strictly descending chain with respect to a well-quasi-ordering [21]. The algorithm therefore always terminates, and $\mathbf{m}$ is reachable from $\mathbf{m_0}$ in the root GVASS $\mathcal{G}$ if and only if some GVASS in the tree satisfies $\theta$.

**The $\boldsymbol{\theta}$ Condition** Kosaraju's main predicate comprises two parts. $\theta_1$ is a global property of the system, while $\theta_2$ must hold for each component.

$\theta_1$ : There exist *pseudo-runs* through the GVASS which use every edge in every component unboundedly many times, and attain unboundedly large values for every unconstrained coordinate (here a *pseudo-run* is a run over $\mathbb{Z}^d$ rather than over $\mathbb{N}^d$). This condition can be formulated as an integer linear programming problem. From this we obtain a semilinear set of vectors of variables representing counts of transition occurrences and values of unconstrained coordinates. If there is no bounded variable then $\theta_1$ holds. Otherwise, one such bounded variable is "refined" by either constraining the associated coordinate's value or by unfolding the associated transition. For example, we may deduce that the number of firings of $t_0$ never exceeds 1 in our example GVASS $\mathcal{G}_{\text{ex}}$; we generate the refinements as in Figure 2.

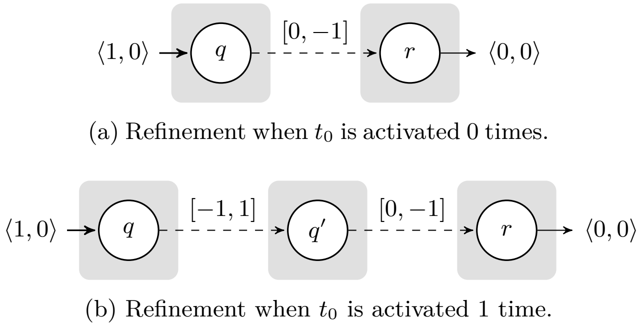

Fig. 2: Refinement of $\mathcal{G}_{\text{ex}}$ by removing bounded transition $t_0$.

$\theta_2$ : Each non-trivial component of the GVASS contains some path from the initial to the final state, via which all unconstrained coordinates are increased. The same must also hold if the component's arcs are reversed. We can evaluate $\theta_2$ using standard algorithms which compute the coverability set. If $\theta_2$ fails, then some coordinate is bounded everywhere in the state space of the component; such a coordinate is refined by removing it entirely (making it *rigid*) and encoding its possible values into the component's states.

**Solving Coverability** We are able to reduce from the coverability problem to the reachability problem in the following way. Suppose we are intending to *cover* some vector $\mathbf{m}$—that is, we wish to reach any vector $\mathbf{m}'$ such that $\mathbf{m}' \geq \mathbf{m}$. We introduce a new state $\mathit{\Delta}$, and add a transition $\delta$ from the final state of the original VASS to $\mathit{\Delta}$, which subtracts $\mathbf{m}$ on activation. As $\mathit{\Delta}$ can only be reached by subtracting $\mathbf{m}$ from our current vector, reaching a vector $\mathbf{m}' \geq \mathbf{m}$ (i.e. covering $\mathbf{m}$) is equivalent to reaching state $\mathit{\Delta}$. For each vector coordinate we introduce a looping transition on $\mathit{\Delta}$ which reduces the value of that coordinate by 1. This ensures that $(0, ..., 0)$ can be reached from any configuration in state $\mathit{\Delta}$. As a result, covering $\mathbf{m}$ in the original net is equivalent to reaching $(\mathit{\Delta}, (0, ..., 0))$ in the augmented version.

**Implementation** KREACH is implemented in the Haskell programming language. This is a strongly-typed, functional language with lazy evaluation. The language was chosen for its high level of expressiveness, type-safety, and the ease of translation between algorithm and implementation.

We represent the algorithm as a function which takes a list of GVASSs, and returns a `KosrajuResult`. We perform a depth-first search of the refinement tree, either finding a refinement which permits reachability (`KosarajuHolds`) or exhausting all possibilities (`KosarajuDoesNotHold`). The algorithm is guaranteed to terminate [17], and so constitutes a full decision procedure for reachability.

The ILP subproblem ($\theta_1$) is solved with the `SBV` (SMT Based Verification) package, an interface to a variety of SMT solvers. We formulate all the constraints as an integer linear program, and evaluate with the `ldn` function (Linear Diophantine equations over Naturals).

The coverability subproblem ($\theta_2$) is solved by an implementation of the standard Karp-Miller algorithm for Vector Addition Systems [16]. This algorithm computes the *coverability set*—the upward closure of the set of all vectors that are reachable in a net from some starting vector. The extensible nature of the code allows the basic implementation to be swapped out for a more optimised one (e.g. based on [10]) at a later stage.

We ensure that the strongly connected property holds by decomposing the original GVASS via the SCC implementation found in the `Data.Graph` module.

**Optimisations** In spite of the ominous non-elementary complexity lower bound, some effort was still undertaken to improve the runtime of test cases. A number of minor improvements have been made over the standard algorithm which remove unneccessary computations.

For example, when constructing refinements for a GVASS $\mathcal{G}$, when a variable is bounded above by some constant $c$, Kosaraju suggests to generate refinements $R_i(\mathcal{G})$ for every $i$ from $\{0, \ldots, c\}$. Instead, we refine only to $R_i(\mathcal{G})$ for values $i$ that feature in the corresponding semilinear set.

The algorithm has also been multithreaded with Haskell's lightweight concurrency toolkit [1], so that it evaluates refinements in parallel rather than sequentially. Any return value of `KosarajuHolds` will terminate the program.

The program uses the `vass` library (released as part of this publication) to parse file formats. By default a parser for MIST's `.spec` format[1] is provided. This format is traditionally a representation of coverability problems; KReach translates these to reachability problems by replacing $p \geq n$ constraints by $p = n$ in target places.

## 3 Installation and Usage

**Installation** The KReach tool is available from a public GitHub repository. One can clone the repository in full with the following command:

```
git clone https://github.com/dixonary/kosaraju.git
```

The program is built against the Haskell `stack` toolchain[2]. In order to build the tool, a locally installed version of `stack` is required. The tool can be compiled and locally installed by running `stack install` in the cloned directory. One must also ensure that an SMT solver is installed and accessible on the user's binary path; `z3`[3] and `cvc4`[4] are supported. A compiled program binary, along with benchmarks, is provided on the "Releases" section of the GitHub page.

**Usage** The compiled `kosaraju` tool can be interacted with through the command line. Simple wrapper scripts are provided; the standard invocation is `kreach FILENAME` to check reachability, and `kcover FILENAME` for coverability. Intermediate output can be hidden by providing the `-q` (quiet) flag.

Figure 3b shows the relative performance of `z3` against `cvc4` for growing inputs. `cvc4` tends to far outperform `z3` on the constructed ILP problems.

## 4 Experimental Results

KCover allows us to use benchmarks for the coverability problem as a source of test cases for the reachability algorithm. The suite provided with the tool includes also a number of test cases for various aspects of the implementation, as well as examples from the non-elementary lower bound construction [7].

`KReach` was evaluated against many problems and solvers from the literature on coverability. `QCover` [4] implements coverability based on relaxation to continuous coverability; `ICover` [11] refines this further with inductive invariants.

1 https://github.com/pierreganty/mist/wiki
2 https://docs.haskellstack.org/en/stable/README/
3 https://github.com/Z3Prover/z3
4 https://cvc4.github.io

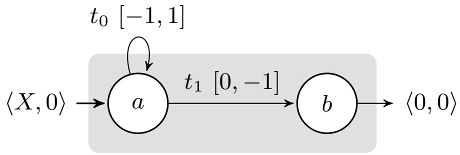

(a) The parameterized version of our original sample case $\mathcal{G}_{ex}$, notated $\mathcal{G}_{ex}(X)$.

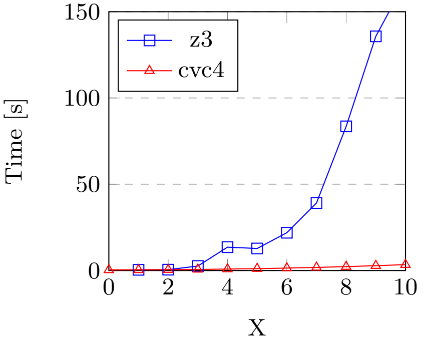

(b) Time against Parameter X for $\mathcal{G}_{ex}(X)$ with the supported solvers.

Table 1 includes some specific instances which are representative of the broader trends in experimental results. On many safe cases, such as `Kanban` and `Bingham`, KReach is able to determine safety faster than state of the art coverability solvers by finding zero valid refinements (terminating the search immediately). On some safe nets such as `Manufacturing`, KReach cannot immediately rule out coverability in this way, and the refinement tree must be explored. The `Bug_Tracking` examples induced intractably large ILP problems. Unsafe cases such as `PNCSACover` induced large refinement trees, which were unable to be explored fully within the time limit.

| Instance | Outcome | MIST (s) | Qcover (s) | Icover (s) | **KReach** (s) |
|---|---|---|---|---|---|
| Kanban | safe | 404 | TLE | TLE | 1 |
| Bingham_h150 | safe | TLE | TLE | TLE | 533 |
| Manufacturing | safe | 1 | 0 | 0 | 4 |
| Bug_Tracking_x0 | safe | MLE | 13 | 33 | TLE |
| PNCSACover | unsafe | 3 | 27 | 59 | TLE |

Table 1: Sample of test cases. All results were computed on consumer hardware. MLE = Memory Limit Exceeded (4GB); TLE = Time Limit Exceeded (1 hour).

## 5 Concluding Remarks

The experimental results suggest that KReach may be a fruitful source of static invariants for ruling out coverability on some classes of Petri nets. One line of further work may be to attempt to formally classify those nets for which Kosaraju's algorithm is effective in practice.

Further work may also include optimisations based on the novel theoretical developments in the Ackermannian upper bound proof [21], and building parsers to enable experiments on instances of problems that are known to reduce to reachability in Petri nets (e.g., in logic [15,8], concurrent systems [9] or process calculi [25]).

**Data Availability Statement** The data analyzed here are available in the Figshare data repository: https://doi.org/10.6084/m9.figshare.11887956
